# Case Report: Microangiopathic Hemolytic Anemia With Normal ADAMTS13 Activity

**DOI:** 10.3389/fmed.2021.589423

**Published:** 2021-03-02

**Authors:** Nicola Osti, Greta Beschin, Marzia Goldin, Lucia Guidolin, Enrico Panero, Alice Sartori, Alice Parisi, Maurizio Cantini, Francesca Pizzolo, Oliviero Olivieri, Simonetta Friso

**Affiliations:** ^1^Department of Medicine, School of Medicine, University of Verona, Verona, Italy; ^2^Department of Diagnostics and Public Health, School of Medicine, University of Verona, Verona, Italy; ^3^Department of Transfusion Medicine, University Hospital, Verona, Italy

**Keywords:** thrombotic microangiopathy, breast cancer, ADAMTS13, thrombotic thrombocytopenic purpura, disseminated intravascular coagulation, hemolytic anemia

## Abstract

Thrombotic microangiopathies (TMAs) include a heterogeneous group of diseases characterized by abnormalities in the vessel walls of arterioles and capillaries resulting in microvascular thrombosis that typically presents with a microangiopathic hemolytic anemia (MAHA) and severe thrombocytopenia. We describe here the case of an 82-year-old woman, who came to our attention for a clinical condition consistent with thrombotic microangiopathy. Even if initially highly suggestive for a thrombotic thrombocytopenic purpura (TTP), the elevated ADAMTS13 activity together with the alteration of the main coagulation parameters (D-dimer elevation, fibrinogen consumption, slightly prolonged prothrombin time), induced us to consider several other diseases in the differential diagnostic process. The case evolved toward a suspected overlapped secondary hemophagocytic syndrome, though the hyperferritinemia was finally interpreted within the frame of a cytokine storm. After a complex diagnostic workup, the clinical and biochemical parameters guided us toward the diagnosis of a cancer-related microangiopathic hemolytic anemia (CR-MAHA) secondary to a relapsing breast cancer with multiple metastatic localizations. Prednisone 1 mg/kg body weight was started, and several units of fresh frozen plasma were infused, obtaining a good control of the hemolysis. No specific oncological therapies were, however, possible, due to the older age and the critically compromised general condition of the patient; therefore, after clinical stabilization, the patient was discharged for treatment in a palliative care Hospital.

## Introduction

Cancer-related microangiopathic hemolytic anemia (CR-MAHA) is a rare pathologic condition and often represents a very challenging enigma for the physician at the frontline while facing a critically ill patient. A broad and fast differential diagnostic process is mandatory to achieve the correct diagnosis for the most appropriate and potentially successful treatment.

The present case prompted us to review the pathophysiology of thrombotic microangiopathies and the most recent literature on this multifaceted cluster of diseases (1, 2). In describing this case, we have an intent of highlighting how crucial it is to dedicate extreme attention to the several coagulative and hemachrome parameters for the achievement of a correct diagnosis of such complex pathologies.

Written informed consent was obtained from the patient for the publication of this case report.

## Case Description

An 82-year-old woman was referred in December 2019 to our University Hospital Internal Medicine Unit with a diagnosis of thrombotic thrombocytopenic purpura, from a nearby community hospital where she had been admitted few days before for an episode of mild epistaxis.

No neurological or major hemorrhagic/thrombotic events were reported. From collection of her clinical history emerged a type II diabetes mellitus treated with metformin and a history of ductal infiltrating breast cancer. The breast carcinoma had been diagnosed in 2006 and firstly treated with quadrantectomy and radiotherapy (QUART) and tamoxifen and, after documented relapse in April 2019, with radical mastectomy and axillary lymph nodes dissection after which she restarted hormone therapy with anastrazole. The most recent follow-up tests were negative for disease relapse. As shown in Table 1, when she was admitted her blood tests showed a mild normochromic normocytic anemia (Hb 9.3 g/dL) with increased reticulocyte count (325,000 per μl), and a severe thrombocytopenia (platelet count 32,000 per μl); lactate dehydrogenase (LDH) was increased (1200 U/L) as it was total bilirubin (2.5 mg/dL). Coagulation tests highlighted a slightly prolonged prothrombin time (INR 1.4) with a normal activated partial thromboplastin time and a severely reduced plasma concentration of fibrinogen (0.5 g/L) and markedly elevated plasma concentrations of D-dimer (7000 U/L). Direct and indirect antiglobulin tests were negative. Renal function indexes and serum electrolytes concentrations were within the normal range. A peripheral blood smear showed the presence of an elevated number of schistocytes (about 20%).

**Table 1:** Biochemical parameters at the time of admission and dismission from the Internal Medicine Unit.

**Biochemical parameters**	**At admission**	**At dismission**
Hemoglobin (g/dl)	9.3	11.3
Mean corpuscular volume (fl)	96	94
Platelets count (per μl)	32,000	118,000
Reticulocyte count (per μl)	325,000	–
Total bilirubin (μmol/liter)	80	16
LDH (U/liter)	1200	872
Creatinine (μmol/liter)	96.8	57.2
Aptoglobin (g/liter)	<0.08	–
D-dimer (ng/ml)	800	–
INR	1.42	1.18
aPTT	1.07	1.03
Fibrinogen (g/liter)	0.5	1.57
ADAMTS13 activity (%)	104	101
Direct Coombs test	Negative	–
Indirect Coombs test	Negative	–
Blood smear for schistocytes count	20%	–

Given the diagnosis of thrombotic thrombocytopenic purpura hypothesized the day prior to referral to our Internal Medicine Unit, the patient had been infused with 800 cc of fresh frozen plasma (FFP).

Right after admission to our Internal Medicine Unit, a sample for ADAMTS13 activity detection was obtained, whose results came to our attention few hours after admission and showed a normal activity (104%).

Even if PLASMIC score, that is, a score that predicts ADAMTS13 deficiency, in suspected thrombotic thrombocytopenic purpura (TTP) with high discrimination was high-intermediate, several aspects did not fit with a presumptive diagnosis of TTP, which was therefore put into doubt. In fact, the markedly reduced fibrinogen and the elevated plasma D-dimer concentrations, together with the normal ADAMTS13 activity, even if taking into account that the patient had undergone FFP infusion before testing are not typical of TTP. Plasma exchange (PEX) was, therefore, not performed, but oral therapy with prednisone 1 mg/kg body weight was started.

Considering also, in differential diagnosis, the hypothesis of a subacute disseminated intravascular coagulation (DIC), even if not fulfiling all the criteria, FFP infusion at low dosage (10 mL/kg/day) was started. Fresh frozen plasma was performed for four consecutive days and then, because of the normalization of PT-INR and aPTT with persistently reduced fibrinogen levels (<1 g/L), fibrinogen infusions were started and maintained for 1 week and eventually stopped, given the absence of either major or minor bleeding events.

To make a correct differential diagnosis of Coombs negative hemolytic anemias, other diagnostic hypotheses such as sepsis, endocarditis, and rheumatic diseases (*in primis* antiphospholipid antibody syndrome and systemic lupus erythematosus), paroxysmal nocturnal hemoglobinuria and folate/vitamin B12 deficiency needed to be excluded. Other primary thrombotic microangiopathies were not clinically probable, given the advanced age of the patient, the absence of prodromal diarrhea, and the normal renal function.

ADAMTS13 activity was subsequently repeated after 1 week, and it was confirmed to be normal. Also, ADAMTS13 inhibitors resulted not detectable.

Remarkably, elevated serum ferritin concentrations were observed (2900 μg/L) and therefore, given the presence of persistently reduced fibrinogen levels, elevated natural killer (NK) cells count (>1000 per μl, even if it is clearly not the right test to demonstrate an altered NK cells function), a bone marrow biopsy was performed showing a certain grade of hemophagocytic phenomenon.

**Figure 1:**
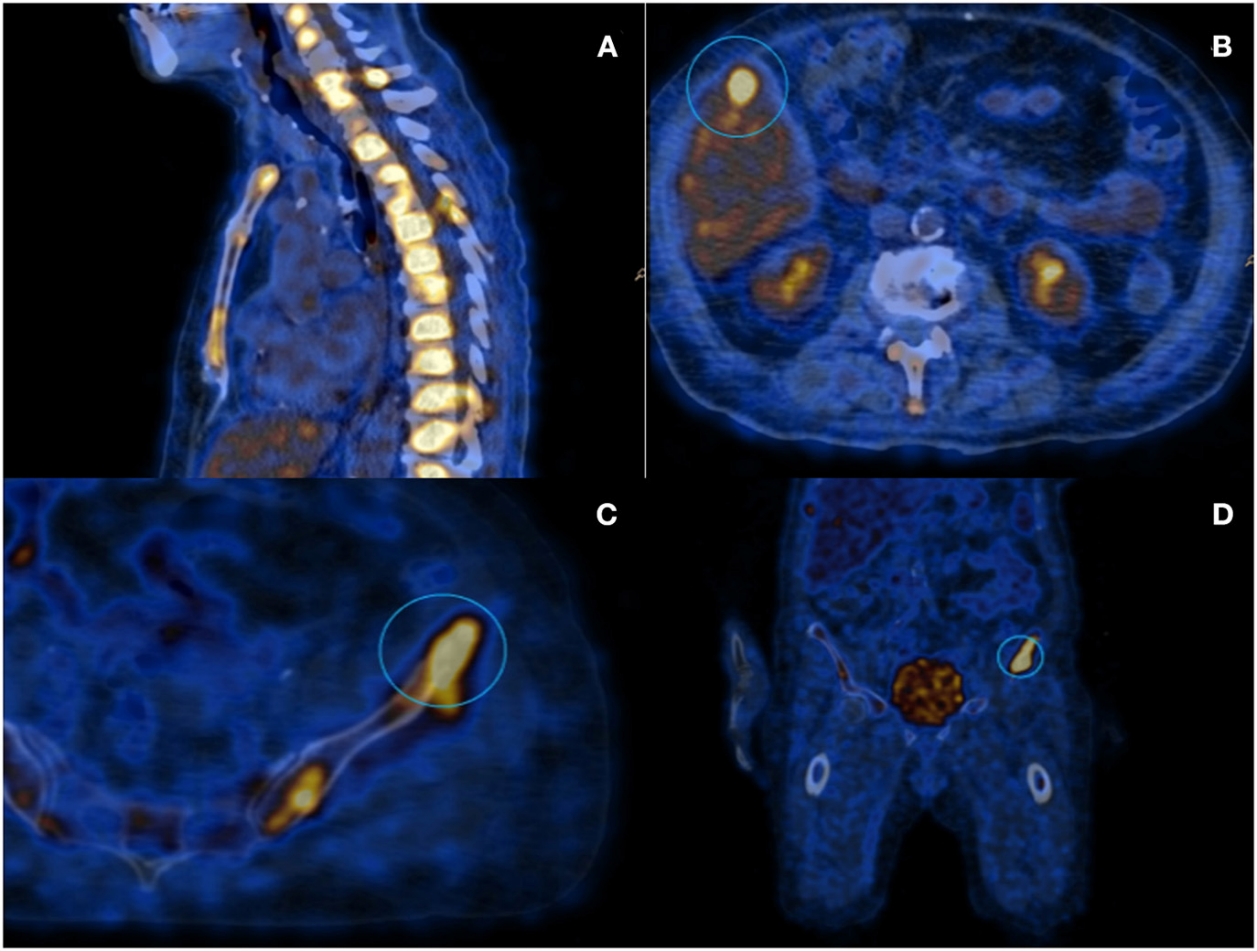
PET scanning with 18-FDG: **(A)** Image showing diffuse hypermetabolism located at the spinal column; **(B)** Hepatic lesion of about 1 cm diameter; and **(C,D)** Iliac lesion that underwent to CT-guided needle-biopsy.

Even if neoplastic markers, including Ca-15.3, were normal, concerns arose for the presence of a diffuse relapse of breast carcinoma, and therefore scar and bilateral axillary ultrasonography, along with contralateral mammography, were performed, all of which were negative for neoplastic localizations. A PET/CT scan was also performed, which showed instead a diffuse fludeoxyglucose hypercaptation in almost all vertebral bodies together with other multiple focal bone lesions, and a unique hepatic lesion of about 1 cm diameter (Figure 1).

**Figure 2:**
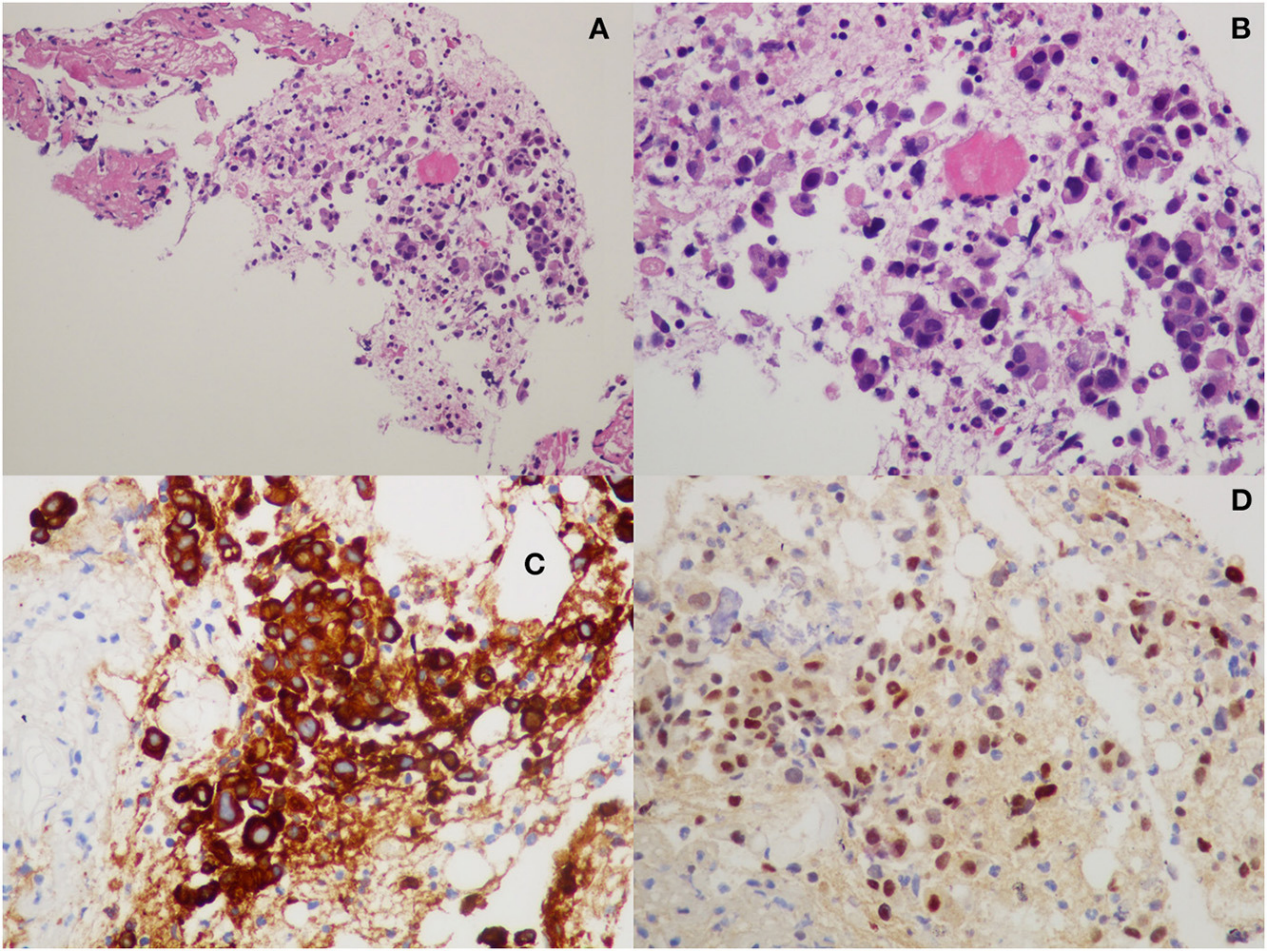
Histopathological examination from iliac bone biopsy: **(A,B)** hematoxylin-eosin staining; **(C)** cytokeratin-8 immunohistochemistry; and **(D)** GATA-3 immunohistochemistry.

The hepatic lesion was too small to possibly perform a US-guided biopsy; therefore, with the support of our interventional radiologist, we decided to perform a CT-guided needle biopsy of the left iliac crest (Figures 1C,D), which resulted positive for the presence of neoplastic dedifferentiated cells expressing keratins 8-18-19 and GATA-3 at immunohistochemistry, compatible with metastatic breast carcinoma localizations (Figure 2).

The global performance status of the patient progressively and rapidly declined during the period of hospitalization, precluding the possibility of specific oncological therapies; hence, the patient was lastly dismissed to another hospital for palliative care, maintaining low dose of oral prednisone therapy (Table 1).

## Differential Diagnosis

When approaching thrombotic microangiopathy (TMA) syndromes, terminology is a key element to understand the entire diagnostic process. Microangiopathic hemolytic anemia (MAHA) is a descriptive term for non-immune hemolysis (Coombs-negative hemolysis) resulting from intravascular red blood cell fragmentation that produces schistocytes on the peripheral blood smear where characteristic laboratory data include increased serum LDH, increased indirect bilirubin, and low serum haptoglobin (1, 2).

Thrombotic microangiopathies describes a specific pathologic lesion in which abnormalities in the vessel wall of arterioles and capillaries lead to microvascular thrombosis. Not all MAHA are caused by a TMA, but nearly all TMAs cause MAHA and thrombocytopenia (1, 2).

Thrombotic microangiopathies is a pathologic diagnosis made by tissue biopsy, typically a kidney biopsy; however, it is commonly inferred from the observation of MAHA and thrombocytopenia in the appropriate clinical setting.

Primary TMA syndromes include TTP (hereditary or acquired), Shiga toxin-mediated haemolytic uremic syndrome (ST-HUS), drug-induced TMA (DITMA) syndromes, complement-mediated HUS (hereditary or acquired), and rare hereditary disorders of vitamin B12 metabolism or factors involved in hemostasis (1).

As for the case of our patient, the presence of a microangiopathic hemolytic anemia and thrombocytopenia is, at a first glance, indicative of TTP.

However, the normal ADAMTS13 activity is a key element to exclude TTP, in which it is almost always below 10% (3, 4). Complement-mediated hemolytic uremic syndrome (HUS) typically presents with an impaired renal function, and so does Shiga-toxin-associated HUS; in the latter case, TMA is usually preceded by diarrhea, abdominal pain, and fever. No new drugs had been recently introduced by the patient, so even the diagnosis of a drug-induced TMA was unlikely.

In megaloblastic anemia, ineffective erythropoiesis mimics MAHA, because of the elevated indirect bilirubin, elevated LDH, and reduced aptoglobin concentrations. In such a case, the increased MCV is the key factor to guide the correct diagnosis. Thrombocytopenia and leucopenia are also generally present, but the degree of thrombocytopenia is usually not as severe as in TTP. Dosage of serum vitamin B12 plus autoantibodies to intrinsic factor in the case of pernicious anemia and folate concentration are then confirmatory.

Hemolytic anemia in Evans syndrome and antiphospholipid antibody syndrome is not due to microangiopathy, and its pathogenesis is invariably autoimmune then featuring a Coombs-positive hemolytic anemia.

In the case of paroxysmal nocturnal hemoglobinuria, in which a Coombs-negative hemolytic anemia is present, thrombocytopenia is due to a reduced medullary production in the context of an aplastic anemia rather than a peripheral consumption; in such a case the diagnosis is confirmed by flow-cytometry for glycosylphosphatidylinositol (GPI)-linked proteins is the confirmatory test.

Moreover, in TTP, clot formation is very rarely dependent by the coagulation cascade, being the clot is almost entirely built of platelets and the von Willebrand factor (vWF). The prolonged PT-INR, the reduced fibrinogen, and the elevation of D-dimer plasma levels are suggestive of DIC, but DIC is not generally associated with the presence of schistocytes in the peripheral blood smear (5). Moreover, in this case, the antithrombin-III (AT-III) levels were normal, making the differential diagnosis even more challenging.

Taking into consideration all of these biochemical parameters, together with the diagnosis of metastatic breast cancer relapse, we think that the diagnosis of cancer-related microangiopathic hemolytic anemia (CR-MAHA) is the most appropriate to solve the case.

Finally, some case reports of hemophagocytic syndrome presenting as thrombotic microangiopathy are reported in the literature, and the possible overlap even of a secondary form of hemophagocytic syndrome made really hard the right interpretation of this aspect (6, 7).

The elevated ferritin levels were interpreted as the epiphenomenon of a cytokine storm, a very complex life-threatening systemic inflammatory syndrome involving elevated blood concentration of cytokines and a hyperactivation of immune cells triggered by several clinical conditions, such as monogenic and autoimmune disorders, among which are *in-primis* hemophagocytic lympho-histiocytosis (HLH), infectious diseases (including COVID-19), autoimmune pathologies, various therapies, and even cancers (8). In the case described here, the hemophagocytic phenomena observed at the bone marrow biopsy were very limited, and in the absence of more specific tests, such as the dosage of soluble CD25 and the evaluation of NK cells activity, the diagnosis of a secondary HLH could not be formulated with absolute confidence.

## Discussion and Literature Review

Endothelial dysfunction in cancer is an established event (9), and CR-MAHA, especially in cases of widespread metastasis of a malignant tumor, is a rare but well-described condition (10). Cancer-related microangiopathic hemolytic anemia has been reported in association with several types of carcinomas as observed in a report of 168 cases of CR-MAHA, where gastric cancer was the most frequent, followed by breast, prostate, and lung cancer (10, 11). Even if CR-MAHA in breast cancer does not seem to be associated with a specific histological type, ductal infiltrating carcinoma is the most commonly reported (10). The pathophysiology behind MAHA in malignancy is yet not well-understood, even if it is thought to be rather a paraneoplastic syndrome than an independent condition co-existing with cancer disease. Two mechanisms have been proposed: red cell fragmentation against tumor emboli within blood vessels and an increased vWF multimers release from bone marrow (the latter in response to altered angiogenesis and secondary myelofibrosis) (12). Cancer-related microangiopathic hemolytic anemia must not be confused with chemotherapy-induced MAHA, which is a specific form of drug-induced TMA, particularly frequent when using gemcitabine and mitomycin, and can be both immune and non-immune mediated.

In a 2011 case of a series of eight breast-cancer associated TMAs, ADAMTS13 activity was normal or slightly reduced, and basic coagulation parameters (PT-INR and aPTT) were only minimally altered, exactly as in the case of our patient (13). von Willebrand factor levels were elevated, but it is known that vWF is also an acute phase reactant and that vWF plasma levels are elevated in case of inflammation and many other physical stressful conditions.

The most effective treatment in CR-MAHA is cancer-specific chemotherapy, while PEX is almost useless, and the prognosis is generally very poor (10). Similarly, treatment of secondary hemophagocytic syndrome is based on the specific oncologic treatment, which was unfortunately not possible for our patient due to a degraded performance status, so the choice was directed toward an adequate palliative care.

## Conclusions

We presented the case of an 82-year-old woman with CR-MAHA secondary to a metastatic breast cancer relapse. The main clinical challenge related to this case was to collect in a unifying diagnosis the ample and diverse set of all symptoms and laboratory findings presented by the patient together with the urgency of a prompt diagnosis to decide the most appropriate therapy for a critically ill patient.

## Data Availability Statement

The datasets presented in this article are not readily available because this is a case report and a dataset has not been generated. Requests to access the datasets should be directed to simonetta.friso@univr.it.

## Ethics Statement

Written informed consent was obtained from the individual for the publication of any potentially identifiable images or data included in this article.

## Author Contributions

NO and SF conceived the manuscript, performed analysis and interpretation of clinical data, and wrote the manuscript. GB, MG, LG, EP, AS, AP, MC, FP, and OO performed interpretation of clinical data and contributed to manuscript writing. All authors contributed to the article and approved the submitted version.

## Conflict of Interest

The authors declare that the research was conducted in the absence of any commercial or financial relationships that could be construed as a potential conflict of interest.

## Abbreviations

MAHA: microangiopathic hemolytic anemia
TTP: thrombotic thrombocytopenic
ADAMTS13: a disintegrin and metalloproteinase with a thrombospondin type 1 motif, member 13
QUART: quadrandectomy and radiotherapy
DIC: disseminated intravascular coagulation
PT: prothrombin time
aPTT: activated partial thromboplastin time
INR: international normalized ratio
MCV: mean corpuscular volume
vWF: von Willebrand factor
FFP: fresh frozen plasma
PEX: plasma exchange
PNH: paroxysmal nocturnal hemoglobinuria
GPI: glycosylphosphatidylinositol
NK: natural killer
PET: positron emission tomography
FDG: fludeoxyglucose
US: ultrasonography
CT: computed tomography
CD: cluster of differentiation
LDH: lactate dehydrogenase.
